# Prophylactic nimodipine treatment improves hearing outcome after vestibular schwannoma surgery in men: a subgroup analysis of a randomized multicenter phase III trial

**DOI:** 10.1007/s10143-020-01368-2

**Published:** 2020-08-22

**Authors:** Christian Scheller, Stefan Rampp, Sandra Leisz, Marcos Tatagiba, Alireza Gharabaghi, Kristofer F. Ramina, Oliver Ganslandt, Cordula Matthies, Thomas Westermaier, Gregor Antoniadis, Maria T. Pedro, Veit Rohde, Kajetan von Eckardstein, Konstanze Scheller, Christian Strauss

**Affiliations:** 1 Department of Neurosurgery, Martin Luther University Halle-Wittenberg, Ernst-Grube-Str. 40, 06097 Halle (Salle), Germany; 2 Department of Neurosurgery, University of Tübingen, Tübingen, Germany; 3 Department of Neurosurgery, Stuttgart Hospital, Stuttgart, Germany; 4 Department of Neurosurgery, University of Würzburg, Würzburg, Germany; 5 Department of Neurosurgery, University of Ulm, Ulm, Germany; 6 Department of Neurosurgery, University of Göttingen, Göttingen, Germany; 7 Department of Neurosurgery, Westpfalz-Klinikum Kaiserslautern, Kaiserslautern, Germany; 8 Department of Oral and Maxillofacial and Facial Plastic Surgery, Martin Luther University Halle-Wittenberg, Halle, Germany

**Keywords:** Nimodipine, Men, Gender, Hearing preservation, Vestibular schwannoma surgery, Nerve protection

## Abstract

A 2016 published randomized multicenter phase III trial of prophylactic nimodipine treatment in vestibular schwannoma surgery showed only a tendency for higher hearing preservation rates in the treatment group. Gender was not included in statistical analysis at that time. A retrospective analysis of the trial considering gender, preoperative hearing, and nimodipine treatment was performed. The treatment group received parenteral nimodipine from the day before surgery until the seventh postoperative day. The control group was not treated prophylactically. Cochlear nerve function was determined by pure-tone audiometry with speech discrimination preoperatively, during in-patient care, and 1 year after surgery and classified according to the Gardner-Robertson grading scale (GR). Logistic regression analysis showed a statistically significant effect for higher hearing preservation rates (pre- and postoperative GR 1–4) in 40 men comparing the treatment (*n* = 21) and the control (*n* = 19) groups (*p* = 0.028), but not in 54 women comparing 27 women in both groups (*p* = 0.077). The results were also statistically significant for preservation of postoperative hearing with pre- and postoperative GR 1–3 (*p* = 0.024). There were no differences in tumor sizes between the treatment and the control groups in men, whereas statistically significant larger tumors were observed in the female treatment group compared with the female control group. Prophylactic nimodipine is safe, and an effect for hearing preservation in 40 men with preoperative hearing ability of GR 1–4 was shown in this retrospective investigation. The imbalance in tumor size with larger tumors in females of the treatment group may falsely suggest a gender-related effect. Further investigations are recommended to clarify whether gender has impact on nimodipine’s efficacy.

## Introduction

### Scientific background and explanation of rationale

There are ongoing discussions about the best management of sporadic vestibular schwannomas (VS) particularly regarding hearing preservation. Unilateral hearing loss is associated with severe deterioration of quality of life with impaired speech recognition in noise, lack of directionality of sound, and daily fatigue [27]. In principle, possible options are wait and scan, radiotherapy, and microsurgery. Sughrue et al. analyzed 34 articles involving 982 patients with untreated sporadic VS. The overall hearing preservation rate was 54%, which was significantly associated with tumor growth rates [26]. Coughlin et al. reported poor long-term hearing preservation rates after radiotherapy with approximately 80% hearing preservation rate at 2 years posttreatment and approximately 23% after 10 years [3]. However, reported hearing preservation rates after microsurgery vary considerably between 2 and 93% [12]. Therefore, predictive factors for hearing preservation after microsurgical VS removal are important in particular for patient counseling and decision-making. Samii and Matthies reported the following predictive factors for hearing preservation after VS surgery in a series of 1000 cases: small to medium tumor size, good to moderate hearing, short duration of hypoacusis or vestibular disturbances, and male gender with chances of hearing preservation between 47 and 88% [21]. Nadol JB Jr. et al. also found in a series of 144 patients that male sex, smaller tumor size, and higher preoperative speech discrimination scores were significantly correlated with hearing outcome [16]. However, in other series with 792 and 104 patients, only tumor size and preoperative hearing but not male gender were predictive factors for preserved hearing in VS surgery [19, 20]. A retrospective analysis of 1269 patients with unilateral VS showed significant differences between males and females regarding tumor size and symptoms at the time of diagnosis with larger tumors and higher prevalence of hearing loss in men, whereas women more frequently suffered from dizziness [9].

Excluding the gender-specific characteristics, the previous studies suggest a neuroprotective beneficial effect of the prophylactic nimodipine administration in VS and maxillofacial surgeries [24, 25]. In addition, in vitro studies have shown that the pretreatment with nimodipine leads to reduced cell death of neuroblastoma, neuronal, astrocytic, and Schwann cells after mechanical, oxidative, osmotic, and heat-induced stress [10, 14].

### Specific hypothesis

Considering these divergent findings, an additional, retrospective analysis of the 2016 published multicenter, randomized phase III trial investigating gender-related differences in hearing preservation rates after VS surgery with prophylactic nimodipine treatment was performed [24].

## Material and methods

Regarding the detailed description of applied methods, the authors refer to the published paper on the phase III trial (e.g., sample size, randomization) [24]. The most important information is summarized as follows.

### Trial design

The prospective, open-label, 2-arm, randomized, and multicenter study was conducted in compliance with the principles of the Declaration of Helsinki and Good Clinical Practice guidelines and approved by the German Competent Authority. The study protocol was approved by the Ethics Committee and all local review boards of the participating institutions. All patients granted informed consent prior to inclusion. No changes to methods were performed after the trial was started.

### Participants

Adults from 18 years of age with an indication for VS surgery were included. Reasons for exclusion were contraindications against nimodipine, surgery for recurrent VS, pregnancy and lactation period, neurofibromatosis type 2, inoperability, preoperative facial nerve function grade VI according to the House-Brackmann (HB) grading scale [11], and participation in other clinical trials within the last 30 days.

### Interventions

VS resections were performed by a retrosigmoid approach with neurophysiological monitoring (brainstem auditory evoked potentials, continuous facial nerve electromyography, and direct facial nerve stimulation). Nimodipine (1–2 mg/h; Nimotop®, Bayer, Leverkusen, Germany) was started the day before surgery and was continued until the seventh postoperative day. Individual dosage adjustments of nimodipine are described in the previous publication of the trial [24].

### Outcomes, follow-ups, and blinding

Since there was a trend only for better hearing preservation rates (and not for facial nerve function) [24], only hearing function was considered for evaluation between males and females. Cochlear nerve function was documented preoperatively, during the in-patient stay, and 1 year after surgery. Hearing was determined by pure-tone audiometry with speech discrimination and classified using the Gardner-Robertson (GR) scale [6]. Tumor size (according to the Koos grading system) and extent of resection were evaluated by a blinded neuroradiologist based on axial contrast-enhanced T1-weighted magnetic resonance imaging (MRI) performed preoperatively and 3 months after surgery for the multicenter trial [13].

### Statistical methods

Effect-measure statistics were applied to evaluate risk differences. Results are expressed as probability to retain hearing. We used generalized linear models (GLM) with the binomial distribution and identity link. Due to the low number of patients with tumor sizes of Koos 1 and 4, the analysis was performed in a subgroup of patients with tumor sizes of Koos 2 and 3 (72 patients, see Table 2). A first model included treatment, gender, and tumor size as well as gender-size interactions. A second model additionally included gender-treatment interactions. Finally, for estimation of risk differences in male and female patients, respectively, separate models were calculated with terms for treatment and tumor size. GLM analysis was calculated using R [18].

In addition, hypothesis testing was conducted using chi-square and Student’s *t* test. Influence of different variables on outcome was analyzed using regression analysis (SPSS Statistics version 25, IBM Corp., Armonk, NY, USA).

## Results

### Participant flow and numbers analyzed

**Fig. 1:**
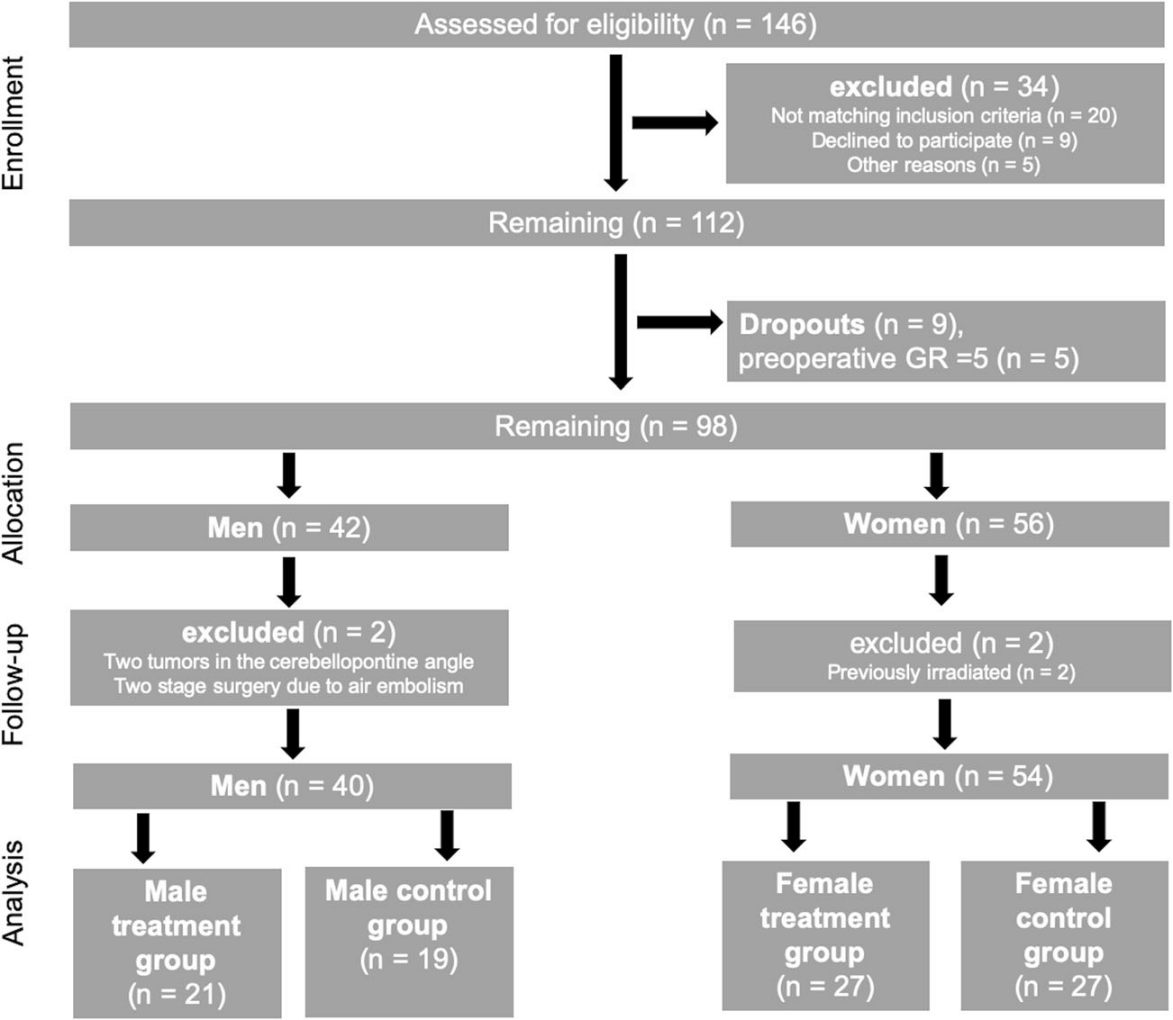
Flow chart

Fourteen of 112 enrolled patients were not suitable for further investigation (9 dropouts and 5 patients with preoperative GR 5), and 4 patients had to be excluded (two tumors in the cerebellopontine angle, two stage surgery, previously irradiated). The primary endpoint of the underlying multicenter study [24] was “facial nerve function 1 year after surgery.” Therefore, patients with preoperative hearing ability of Gardner-Robertson (GR) 5 (deaf) were included. For the present analysis of hearing outcome, patients with preoperative GR 5 had to be excluded. Subsequently, 94 patients (40 men and 54 women) were assigned to the treatment (men: *n* = 21; female: *n* = 27) or to the control group (male: *n* = 19; female: *n* = 27) (Fig. 1).

### Recruitment

The authors refer to the published paper on the phase III trial [24].

### Baseline data

The treatment and the control groups of both males and females were comparable in age, extent of resection, and preoperative cranial nerve function. There was no significant difference regarding tumor sizes between the treatment and the control group in men. In contrast, tumors were significantly larger in the female treatment group as compared with control group (*p* = 0.028). As shown in Table 1, there were 12 women with Koos 2 tumors in the control and only 3 women with Koos 2 tumors in the treatment group. In contrast, larger tumors (Koos 3 and 4) were more frequently observed in the treatment group (14 versus 24 women) (Table 1).

**Table 1:** Baseline data

	Male patients			Female patients			Test
Variable	Control group	Treatment group	*p* value	Control group	Treatment group	*p* value	
Mean age in years ± SD	46.2 (11.5)	48.5 (11.5)	0.903	49.9 (13.2)	48.6 (14.3)	0.587	*t* test
Koos grade (tumor size)			0.670			*0.028*	Chi-square
1	1	0		1	0		
2	8	10		12	3		
3	6	8		10	16		
4	4	3		4	8		
GR class (preoperative hearing)			0.459			0.254	Chi-square
1	6	8		10	10		
2	7	7		7	8		
3	6	4		10	6		
4	0	2		0	3		
5	0	0		0	0		
HB grade (preoperative facial nerve function)			0.168			0.313	Chi-square
I	19	19		26	27		
II	0	2		1	0		
Extent of resection			0.539			0.685	Chi-square
Subtotal 2–10 mm	0	1		4	2		
Residual < 2 mm	1	2		2	2		
Complete	18	18		21	23		

In the female treatment group tumor sizes were significant larger compared to the female control group

### Outcomes and estimation

#### Hearing 1 year after surgery

Effect-measure statistics evaluated risk differences of hearing preservation 1 year after surgery. A first GLM with terms for treatment, gender, tumor size, and gender-tumor size interactions yielded a risk difference of RD = 0.19 (95% confidence interval: − 0.03 to 0.39, *p* = 0.084). Adding a gender-treatment interaction to this model resulted in interaction contrast for hearing preservation RD of IC = 0.31 (− 0.13 to 0.74, *p* = 0.17). Risk differences of hearing preservation with and without treatment were RD = 0.36 (0.04 to 0.64, *p* = 0.016) for male and RD = 0.05 (− 0.27 to 0.34, *p* = 0.74) for female patients.

Logistic regression analysis similarly showed an effect for higher hearing preservation rates (pre- and postoperative GR 1–4 and GR 1–3) in the male treatment (*n* = 21) compared with the male control (*n* = 19) group (*p* = 0.028 and *p* = 0.024, respectively), but not in females comparing 27 women in both groups (*p* = 0.077). Preoperative hearing of GR 1–2 was documented in 28 men (15 in the treatment and 13 in the control group). However, postoperative hearing ability of GR 1–2 was preserved in only five men (three patients in the treatment group). A robust statistical evaluation in men with preoperative GR 1–2 was therefore not possible.

Further subanalysis regarding tumor sizes showed statistically significant higher hearing preservation rates in males with Koos 2 tumors (*p* = 0.030, Table 2). In the male treatment group of Koos 2 tumors, hearing was preserved in 7 of 9 cases, whereas in the male control group of Koos 2 tumors, hearing was preserved in 2 of 8 cases. Regarding tumor sizes of Koos 3 and 4, there was no statistically significant difference. No reliable conclusions can be drawn for Koos 1 tumors (*n* = 1).

**Table 2:** Hearing preservation rates (postoperative GR 1–4) in relation to tumor size in men and women

Tumor size (Koos)	Male patients (*n* = 40)		*p* value (chi-square test)	Female patients (*n* = 54)		*p* value (chi-square test)
	Control group (*n* = 19)	Treatment group (*n =* 21)		Control group (*n* = 27)	Treatment group (*n* = 27)	
1	1/1	0/0	-	1/1	0/0	-
2	2/8	7/9	*0.03*	8/12	2/3	1
3	1/6	3/8	0.393	3/10	6/16	0.861
4	0/4	1/4	0.285	0/4	1/8	0.46

There were no differences observed regarding hearing preservation rates in females for both overall and all Koos subgroups (*p* = 0.709). However, all females with Koos 4 tumors of the control group became deaf, whereas in two of eight (25%), hearing was preserved in the female therapy group. Concerning Koos 2 tumors of females, no statistical evaluation is reasonable since 12 of 15 Koos 2 tumors were randomized to the control group (Table 2).

#### Facial nerve function 1 year after surgery

The GLM with terms for treatment, gender, tumor size, and gender-tumor size interactions yielded a risk difference of RD = 0.02 (95% confidence interval: − 0.16 to 0.20, *p* = 0.81).

Logistic regression analysis showed no gender-related effect and no interaction between nimodipine and gender regarding preservation of facial nerve function. Facial nerve function 1 year after surgery was HB I in 74% of the males and in 69% of the females (*p* = 0.29, chi-square test), HB II in 10% of the males and in 15% of the females, HB III in 12% of the males and in 14% of the females, HB IV in 2% of the males and in 1% of the females, and HB V in 2% of the males and in 1% of the females.

### Adverse effects of treatment (harms)

As already described in previous studies [23, 24], nimodipine was well tolerated, and no drug-induced mortality or serious adverse events were observed. Dose-dependent hypotension resulting in dose reduction or discontinuing of nimodipine was the only relevant side effect [24].

## Discussion

### Limitations

Data from a prospective and randomized multicenter trial were evaluated in a retrospective way, which may result in some selection bias and in a possible overestimation of statistical results. Since tumor size was not a randomization factor in the original study [24], the imbalance regarding tumor sizes between the control and the treatment groups in women is the most relevant limitation for the evaluation of a gender-related effect. However, risk difference and logistic regression analysis showed a treatment effect for higher hearing preservation rates in men, although the confidence intervals for risk differences were rather broad. Considering that tumor size is one of the most reliable predictive factors for hearing preservation [22], it is surprising that there were no differences observed regarding hearing preservation rates in females between the control and the treatment group. This finding may be caused by a neuroprotective nimodipine effect, which however might be obfuscated by an imbalance of tumor sizes and the broad risk difference confidence intervals. In addition, the gender-treatment interaction contrast failed to show a clear gender difference. Therefore, it is possible that nimodipine preserves hearing function in both men and females. An evaluation bias is very unlikely since hearing ability was determined by pure-tone audiometries with speech discrimination and hearing abilities were classified by blinded expert reviewers. However, not only an administered neuroprotective agent but also several additional factors have impact on the hearing ability after VS surgery.

### Generalisability

#### Gender- and nimodipine-related effects in vestibular schwannoma surgery

A professional literature research was performed. The following key words were used: nimodipine, gender, men, women, calcium channel blocker, subarachnoid hemorrhage, pharmacokinetics, sex-related differences, stroke, vestibular schwannoma, vasoactive, and neuroprotection.

Gelmers et al. reported a potential beneficial effect of early nimodipine treatment in acute ischemic strokes, which was limited to men [7].

A gender-related effect has also been discussed in presentation of VS, outcome after VS surgery, and regarding the efficacy of nimodipine treatment.

Samii et al. evaluated 1000 VS surgeries regarding factors influencing the chance of hearing preservation [22]. Advantageous factors were male gender, small to medium tumor sizes, good to moderate hearing, and short duration of hypoacusis or vestibular disturbances. Cadaveric studies showed that the mean lateral angle of the internal acoustic canal is greater in females (first line: medial petrous bone wall; second line: porus to fundus) [8, 15], which may influence the angle of dissection between nerves and tumor and therefore outcome. Nevertheless, Al-Shudifat et al. reported in a retrospective series of 395 patients that female gender and patients over 50 years with larger tumor have a higher risk for reduced work capacity after VS surgery [1]. However, it remained unclear, if there is a correlation between hearing preservation rates and reduced work capacity in women.

Regarding nimodipine’s efficacy, sex-related differences of cytochrome P450 (CYP) 3A4 have been reported. CYP3A4 is responsible for the metabolism of nimodipine [4]. Considering women have higher CYP3A4 content in the liver [2], it may be possible that nimodipine is metabolized faster in women resulting in lower drug levels and reduced efficacy. Accordingly, Futuro-Neto et al. showed that female albino mice needed higher doses of nimodipine for neuroleptic-induced catalepsy than males [5].

Without considering tumor size and drug metabolism, the molecular mechanisms of this putative gender effect are unclear. In vitro*,* the antiapoptotic effect of nimodipine pretreatment is associated with the activation of AKT and CREB by phosphorylation [14]. Pan et al. showed recently in a rat model that the sex-dependent effects of the G protein-coupled estrogen receptor (GPER) activation, which plays a role in neuroprotection, depend on AKT activation [17]. In summary, it could be possible that the estrogen status or ER activation has an influence on the gender-specific nimodipine mode of action. Therefore, the influence of gender and hormone status on the nimodipine neuroprotective effect should be investigated further to shed light on sex differences in nimodipine treatment efficiency and enable a personalized neuroprotective therapy.

### Interpretation

#### Cochlear nerve function 1 year after surgery

Nimodipine’s efficacy was predominantly observed in men with larger differences in patients with Koos 2 tumors. This might be interpreted as a gender-related nimodipine effect; however, statistical analysis did not reveal significant differences. The lack of a treatment effect in women could thus be mainly due to the imbalance of tumor sizes. Since hearing preservation rates are strongly influenced by tumor sizes, nimodipine is obviously more useful in tumor sizes with higher chances for postoperative hearing. This finding was limited to postoperative hearing classes GR 1–4 and GR 1–3, but not to GR 1–2. Due to the small sample size, further studies are needed to clarify if postoperative useful hearing (GR 1-2) also benefits from nimodipine treatment. For Koos 2 tumors in women, a robust statistical evaluation was not possible due to strongly unbalanced distribution with only 3 women in the treatment group.

#### Facial nerve function 1 year after surgery

A benefit from nimodipine for facial nerve outcome was not observed. Considering that in both the female and the male groups’ facial nerve function was excellent or good (HB 1–2) 1 year after surgery in 84%, a potential effect of nimodipine may not be detectable. In contrast to the cochlear nerve, the facial nerve has the potential for regeneration, which may influence the analysis of nimodipine’s efficacy.

## Conclusions

In the presented retrospective analysis, prophylactic nimodipine was safe and preserved hearing predominantly in men with Koos 2 tumors but also in the male overall group. Whether the perioperative administration of nimodipine has also beneficial effects in women has to be clarified by further investigations. A possible gender effect should be investigated in further studies.
